# Evaluation of inflammatory periodontal tissue by detecting the hemoglobin released in gingival crevicular fluid during supportive periodontal therapy

**DOI:** 10.1007/s10266-025-01182-6

**Published:** 2025-08-30

**Authors:** Hiroshi Ito, Yukihiro Numabe, Shuichi Hashimoto, Satoshi Sekino, Etsuko Murakashi, Hitomi Ishiguro, Daisuke Sasaki, Takashi Yaegashi, Hideki Takai, Masaru Mezawa, Yorimasa Ogata, Hisashi Watanabe, Yuichi Izumi, Jun-ichi Kido, Yuka Hiroshima, Toshihiko Nagata

**Affiliations:** 1 Department of Periodontology, School of Life Dentistry at Tokyo, The Nippon Dental University, 1-9-20 Fujimi, Chiyoda-Ku, Tokyo, 102-8159 Japan; 2 The Nippon Dental University, Tokyo, Japan; 3 Department of Conservative, Division of Periodontology, Iwate Medical University School of Dentistry, Iwate, Japan; 4 Department of Periodontology, Nihon University School of Dentistry at Matsudo, Chiba, Japan; 5 Department of Periodontology, Graduate School of Medical and Dental Sciences, Institute of Science Tokyo, Tokyo, Japan; 6 Department of Periodontology and Endodontology, Institute of Biomedical Sciences, Tokushima University Graduate School, Tokushima, Japan

**Keywords:** Gingival crevicular fluid, Hemoglobin, Periodontal examination, Periodontitis, Bleeding on probing

## Abstract

The purpose of this study was to longitudinally investigate the hemoglobin (Hb) test in gingival crevicular fluid of periodontitis patients, together with clinical parameters (plaque index: PlI, probing depth: PD, clinical attachment level: CAL, bleeding on probing: BOP, and gingival index: GI) during two-year supportive periodontal therapy (SPT). A total of 191 periodontal sites in 91 patients during the SPT period were clinically and biochemically evaluated three times (first, second, and third examinations) over two years. After the first examination of the clinical and biochemical parameters, periodontal support treatments were administered and then repeated every three months until the third examination. The values of PD, CAL, GI, and Hb decreased from the first examination to the second or third examination. Notably, the percentage reduction of Hb test values was significant. However, only the BOP value tended to increase. The decreases in these clinical and biochemical parameters suggested that the periodontitis was not exacerbated, but rather tended to recover with periodontal treatment. These reductions in PD, CAL, and Hb values from the first examination were clearly more common in the PD ≥ 5 mm group than in the PD ≤ 4 mm group for all test sites. In this study, the highly sensitive Hb test showed that periodontal treatment for two years suppressed the progression of periodontitis. This trend was significant in the deep gingival sulcus (PD ≥ 5 mm) group, suggesting that the Hb test may be a useful quantitative marker to predict recurrence of periodontitis.

## Introduction

Periodontal disease is one of the infectious diseases caused by oral bacteria. Inflammatory and immune responses due to dental plaque biofilms cause destruction of periodontal tissues, resulting in clinical symptoms such as loss of attachment and alveolar bone resorption [1, 2]. Furthermore, periodontal disease is a highly recurrent disease, and maintenance of high-quality oral hygiene by patients is the cornerstone of periodontal disease prevention and treatment.

First, as a preventive measure, the basic examination for periodontal disease includes measuring probing depth (PD) and checking for bleeding on probing (BOP) with a probe, which requires very delicate technique [3, 4]. In particular, the BOP test is a highly effective test that shows a strong correlation with the outcome of treatments such as scaling and root planing. The test results are also recognized as a factor that can predict the degree of future attachment loss [5]. In addition, the BOP test is considered extremely important because it is used and interpreted appropriately, such as when combined with PD test results to obtain a prognosis for periodontal treatment [6]. However, some reports state the weaknesses and limitations of the BOP test, such as a high negative predictive value compared with the positive predictive value [5] and discrepancies between BOP test results and clinical symptoms [7]. In contrast, periodontal treatment is based on scaling and root planing. The effectiveness of this treatment is thought to be extremely limited, since it depends heavily on tooth morphology, such as pocket depth and root furcation, which indicate the pathological condition of periodontal disease [8, 9].

Given this context, it is necessary to ascertain the state of periodontal tissues as accurately as possible by highly accurate examinations in advance. We believe that it is difficult to obtain standard reproducibility of test results in the conventional measurement of clinical parameters centered on pocket probes due to differences in examiner proficiency [10]. At the same time, probing is a painful test, and depending on the situation, it may be difficult to obtain accurate test results [11]. Even from this perspective, there is a need for a new periodontal disease test.

Therefore, a gingival crevicular fluid (GCF) test, which differs from conventional probing tests because it can be collected painlessly and shows a strong correlation between the component analysis results and the pathology of the collection site, was investigated. First, various biochemical parameters (aspartate aminotransferase, elastase, and protein) were measured in GCF at the SPT stage and compared with those of conventional periodontal tests, including the BOP test [7]. As a result, a large discrepancy was found between the biochemical parameters that suggest tissue damage and the BOP test result, which is one of the clinical parameters. This study showed that the BOP test alone was not highly accurate in detecting inflamed periodontal tissues. Therefore, we believed it was necessary to further explore GCF test markers that can complement the BOP test and detect tissue damage more sensitively.

Previous studies have reported the presence of hemoglobin (Hb) as evidence of bleeding in the GCF of inflamed periodontal pockets [12, 13]. To search in detail for the presence of Hb, one of the biochemical parameters, we measured and evaluated the Hb level in the GCF using immunochromatography (IC) [14]. We found that the Hb test could identify the presence of invisible bleeding in GCF, even when the BOP test was negative [14]. This demonstrated that the Hb test can detect mild tissue damage. In contrast, the same study also found cases in which the BOP test was positive even in pockets without detectable Hb. One reason for these discrepancies is that the BOP test involves inserting a probe into the periodontal pocket and visually assessing tissue vulnerability by checking for bleeding at the bottom of the pocket.

Furthermore, a detailed analysis of periodontal cross-sectional examination results in patients undergoing SPT at the Hb cut-off values for BOP and PD showed that, despite a negative BOP examination, Hb molecules can be detected in the GCF [15]. The Hb level observed in this GCF increased along with other biochemical parameters, such as alkaline phosphatase and protein. This increase in Hb levels strongly suggested the possibility of periodontal disease recurrence. Then, a longitudinal periodontal study, in which biochemical and clinical tests were conducted twice a year, showed that the reduction in Hb levels in GCF due to SPT was associated with a decrease in clinical parameters [16]. These changes in parameters were significantly correlated, except for BOP.

These results demonstrated that the Hb level in GCF could serve as a useful marker to complement clinical parameter tests for periodontal disease examination, such as the BOP test [7, 14–16].

In this study, which is a continuation of a previous study [16], the condition of periodontal tissues during SPT was evaluated continuously over two years. This evaluation used the biochemical parameter (Hb) and the clinical parameters, including the plaque index (PlI), probing depth (PD), clinical attachment level (CAL), gingival index (GI), and BOP. The test results of all parameters used for evaluating periodontal disease were analyzed statistically, and correlations between tests were examined, as well as changes in test values over time.

## Materials and methods

### Experimental design

This two-year observational study, conducted according to the Declaration of Helsinki and STROBE guidelines, built upon our previous research [16]. The research was carried out with approval from the Ethics Committee of Nippon Dental University (No. NDU-T 2017–12) and with the informed consent of the research participants.

### Study population

The criteria for selecting patients were based on our previous study [16]. The starting point was the moment the patient began the SPT phase. Patients with chronic periodontitis who did not smoke, had at least 12 remaining teeth, were in good general health, and received SPT every 3 months were included (Table 1).

**Table 1:** Study population

a. Patient information	
Patients	91
Age, y (mean ± SD)	62.8 ± 10.9
Sex	37 males, 54 females
Inspection site	191

b. Classification of inspection site							
Upper central incisor	42	PD ≤ 4 mm	32	Lower central incisor	18	PD ≤ 4 mm	10
		PD ≥ 5 mm	10			PD ≥ 5 mm	8
Upper lateral incisor	33	PD ≤ 4 mm	22	Lower lateral incisor	21	PD ≤ 4 mm	10
		PD ≥ 5 mm	11			PD ≥ 5 mm	11
Upper canine	29	PD ≤ 4 mm	20	Lower canine	22	PD ≤ 4 mm	12
		PD ≥ 5 mm	9			PD ≥ 5 mm	10
Upper premolar	15	PD ≤ 4 mm	3	Lowerpremolar	11	PD ≤ 4 mm	3
		PD ≥ 5 mm	12			PD ≥ 5 mm	8

c. Classification of periodontitis in patients (n = 91) (Tonetti et al., 2018)												
Stage	I			II			III			IV		
Grade	A	B	C	A	B	C	A	B	C	A	B	C
Localized chronic periodontitis	4	3	1	7	2	–	8	1	–	–	–	–
Generalized chronic periodontitis	–	–	1	2	1	–	44	9	5	–	3	–

a. Since 93 patients dropped out from the first periodontal examination to the third periodontal examination, a comparison of periodontal tissue conditions between the first, second, and third examinations was performed using values (clinical and biochemical parameters) of 191 sites in 91 patients in the third examination. The clinical parameters were PlI, PD, CAL, BOP, and GI. The biochemical parameter was Hb

b. The tooth types at the 191 sites from which GCF was collected are shown in Table 1b. Furthermore, tooth types were classified into PD ≤ 4 mm and PD ≥ 5 mm groups based on the PD from which GCF was collected

c. A total of 91 subjects were classified according to the level of periodontal disease according to the new classification

### Research protocol

**Fig. 1:**
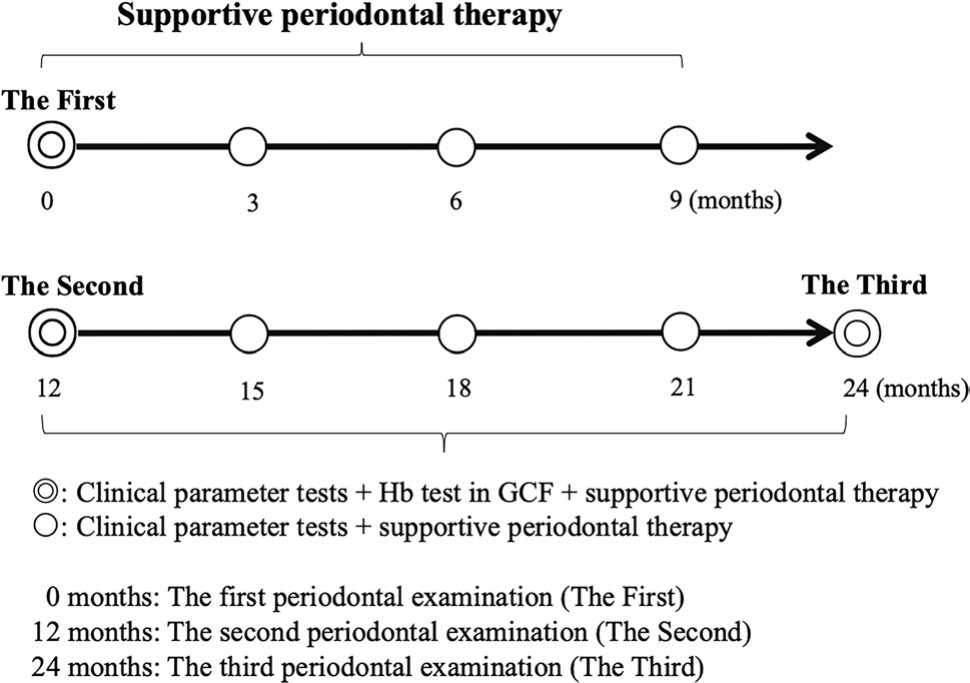
Research protocol. The recall interval is 3 months, and supportive periodontal therapy at each visit includes oral hygiene instruction, professional tooth cleaning, and subgingival debridement using an ultrasonic scaler as needed. The clinical parameter tests are PlI, PD, CAL, GI, and BOP, in that order. The biochemical parameter test is the amount of Hb in GCF. During the first (0 months), second (12 months), and third (24 months) periodontal examinations conducted over two years as part of SPT, GCF samples are collected between the measurements of PlI and PD, and Hb levels measured

This observational study was conducted from 2009 to 2019 (Fig. 1). A comparison was made by conducting measurements of the amount of hemoglobin observed in the periodontal examination and GCF for 191 sites of 91 individuals who did not drop out during the two-year observation period (Table 2). In the three-monthly SPT, oral hygiene instruction and dental cleaning were provided, and subgingival debridement was performed using an ultrasonic device as needed [17]. Hemoglobin levels were measured at baseline (0 months) and at 12 months and 24 months into the SPT.

**Table 2:** Comparisons among the first periodontal examination, second periodontal examination, and third periodontal examination (Steel–Dwass test)

Clinical and biochemical parameter	First periodontal examination	P1	Second periodontal examination	P2	Third periodontal examination	P3
PlI	0.39 ± 0.52	NS	0.29 ± 0.53	NS	0.45 ± 0.64	NS
PD (mm)	3.57 ± 1.79	NS	3.07 ± 1.50	NS	2.95 ± 1.38	*
CAL (mm)	4.88 ± 2.44	NS	3.88 ± 2.20	NS	3.97 ± 2.27	NS
GI	0.82 ± 0.76	NS	0.70 ± 0.75	NS	0.75 ± 0.83	NS
BOP	0.23 ± 0.42	NS	0.26 ± 0.44	NS	0.34 ± 0.47	**
Hb (ng/pocket)	48.16 ± 81.78	**	10.79 ± 32.59	NS	9.71 ± 6.88	**

*P1* First periodontal examination vs second periodontal examination

*P2* Second periodontal examination vs third periodontal examination

*P3* Third periodontal examination vs first periodontal examination

^*^p < 0.05, ^**^p < 0.01

### Measurements of clinical parameters and GCF collection

As per our previous report [16], the periodontal examination was conducted by a periodontist in the following order: PlI [18], GCF collection, PD, CAL, GI [19], and BOP [20, 21]. Pre-calibration by examiners allowed for an error of ± 1 mm in measurements of PD and CAL (n = 10), resulting in a 90% matching rate. The probe used in the examination was the WILLIAMS PROBE (Hu-Friedy Inc., Chicago, IL, USA). Following previous studies, GCF collection was performed using absorbent paper strips (PerioPaper, Oraflow Inc., Plainview, NY, USA) from the pockets of single-rooted anterior teeth in the maxilla and mandible [7, 14–16].

### Measurement of hemoglobin

All GCF samples were subjected to biochemical marker (Hb content) measurement by a biochemist (SH) according to a previously reported method [16]. Briefly, Hb content was measured using an Hb detection kit (Check-Line Hemo, Wakamoto, Tokyo, Japan) that applied the IC method using human monoclonal antibodies. In brief, 10 mL of PBS extract was added to 40 mL of IC solvent containing bovine serum albumin, polysorbate 20 (Tween 20, Sigma-Aldrich, St. Louis, MO, USA), and sodium sulfate in neutral PBS, and Hb in the 50 mL solution was developed using IC paper at 23 °C for 15 min. The IC paper was air-dried overnight, and the amount of human Hb monoclonal antibody labeled with red latex in the chromatogram was measured using a densitometer (GS-800 Calibrated Densitometer PC system, Bio-RAD, Tokyo, Japan) and expressed as ng/pocket [14]. When the amount of Hb observed in each GCF was 1 ng/pocket Hb or more, it was defined as Hb-positive (patent application number: JP2013-257312A).

### Statistical analysis

Statistical analysis was performed according to our previously reported studies [14–16]. First, the distribution of each clinical parameter value and biochemical parameter (Hb) value was tested using the Kolmogorov–Smirnov test. In this study, considering the results that did not follow a normal distribution, multiple comparisons of the clinical parameter values and Hb values at 0 months (baseline), 12 months, and 24 months were conducted using the Steel–Dwass test. Furthermore, the correlations between the values of clinical parameters and Hb were analyzed using Spearman’s correlation coefficient. The statistical software used was SPSS ver. 22.0 J (IBM-SPSS, Inc., Chicago, IL, USA).

Sample size calculation was determined based on the correlation between BOP and Hb test values as the primary endpoint [14]. The power calculation showed that the sample size required to perform this study was n = 32 (correlation coefficient: 0.2, α error: 0.05, power: 0.8). However, at 0 months, more than 50 GCF samples were collected from each facility.

Claffey et al. [22] have already discussed the status of periodontal tissues in SPT patients divided into groups with PD values of ≤ 4 mm and ≥ 5 mm. In this study, the progression of periodontal disease in SPT was thus classified into two groups (PD ≤ 4 mm and PD ≥ 5 mm) and evaluated. Then, within each group, the clinical and biochemical parameter values in the first (0 months), second (12 months), and third (24 months) examinations conducted over two years at one-year intervals were compared over time (Fig. 1).

## Results

### Patients

In the initial periodontal disease examination, 184 patients (73 males, 111 females, average age 63.0 ± 11.3 years) with 401 GCF sampling sites were targeted. At the second periodontal examination one year later, 56 patients dropped out, so 128 patients (53 males, 75 females, mean age 62.4 ± 11.2 years) with 279 GCF sampling sites were included. The 56 patients who were lost to follow-up after one year included 41 victims of the Great East Japan Earthquake, 14 patients who were unable to visit the clinic for personal reasons, and one patient whose target teeth had naturally fallen out. At the third periodontal examination, 37 patients dropped out for personal reasons, leaving 91 patients (37 males and 54 females, mean age 62.8 ± 10.9 years) with 191 GCF sampling sites (Table 1a). Therefore, 191 test sites (Fig. 1) of 91 participants who could be continuously monitored and sampled at the first visit, the second one year later, and the third two years later underwent periodontal examinations.

The breakdown of the 191 sites collected from the 91 patients who were finally included in the analysis was as follows: 21 patients with 1 site, 47 patients with 2 sites, 19 patients with 3 sites, 2 patients with 4 sites, 1 patient with 5 sites, and 1 patient with 6 sites. The tooth types at the 191 sites from which GCF was harvested are shown in Table 1b. Furthermore, the tooth types at the GCF harvest sites in the PD ≤ 4 mm group and the PD ≥ 5 mm group are also shown. Table 1c shows the periodontal disease classifications of the 91 patients [23].

Clinical parameter values and Hb values at the first, second, and third periodontal examinations.

When comparing the changes in periodontal examination values between the first and second visits, the PlI, PD, CAL, and GI values from the first visit decreased at the second visit one year later; only the BOP positivity rate increased. In contrast, the Hb value decreased significantly at the second visit one year later, with a reduction of 77.6% (p < 0.01) (Table 2).

From the first to the third clinical and Hb tests for periodontal disease, the decrease rate of the PD value was 17.4% (p < 0.05), and the BOP value increased from the first test to the third test, with a significant increase of 47.8% (p < 0.01). Meanwhile, the Hb value in the GCF decreased by 79.8% from the first test to the third test (Table 2).

Correlations among all of the clinical parameter values or Hb values at each of the first, second, and third periodontal examinations.

Significant correlations were observed between the clinical parameters and Hb test results of all three periodontal disease tests performed over two years (Table 3a-c).

**Table 3:** Correlations of clinical and biochemical parameters (Abbreviation: CC, coefficient of correlation.)

		PD	CAL	GI	BOP	Hb
a Clinical and biochemical parameters in the first periodontal examination						
PlI	CC	0.354	0.403	0.395	0.170	0.215
	*p* value	0.001	0.001	0.001	0.019	0.003
PD	CC	–	0.845	0.574	0.496	0.383
	*p* value	–	0.001	0.001	0.001	0.001
CAL	CC	–	–	0.546	0.445	0.372
	*p* value	–	–	0.001	0.001	0.001
GI	CC	–	–	–	0.548	0.320
	*p* value	–	–	–	0.001	0.001
BOP	CC	–	–	–	–	0.177
	*p* value	–	–	–	–	0.014
b Clinical and biochemical parameters in the second periodontal examination						
PlI	CC	0.235	0.220	0.419	0.336	0.212
	*p* value	0.001	0.002	0.001	0.001	0.003
PD	CC	–	0.693	0.399	0.464	0.295
	*p* value	–	0.001	0.001	0.001	0.001
CAL	CC	–	–	0.199	0.296	0.195
	*p* value	–	–	0.006	0.001	0.007
GI	CC	–	–	–	0.584	0.352
	*p* value	–	–	–	0.001	0.001
BOP	CC	–	–	–	–	0.246
	*p* value	–	–	–	–	0.001
c Clinical and biochemical parameters in the third periodontal examination						
PlI	CC	0.254	0.192	0.348	0.305	0.217
	*p* value	0.001	0.008	0.001	0.001	0.003
PD	CC	–	0.696	0.453	0.426	0.320
	*p* value	–	0.001	0.001	0.001	0.001
CAL	CC	–	–	0.302	0.301	0.215
	*p* value	–	–	0.001	0.001	0.003
GI	CC	–	–	–	0.762	0.411
	*p* value	–	–	–	0.001	0.001
BOP	CC	–	–	–	–	0.411
	*p* value	–	–	–	–	0.001

Changes in the clinical parameter values or Hb values among the first, second, and third periodontal examinations in the PD ≤ 4 mm group.

The clinical parameters did not show any significant changes from the first periodontal disease examination to the second examination. Although no significant differences were observed, the BOP value increased to 2.3 times the value of the first examination in the second examination. In contrast, the Hb level decreased significantly at the second periodontal examination compared with the first one, with a reduction of 81.0% (p < 0.01) (Table 4).

**Table 4:** Comparison of periodontal examinations at a site of PD ≤ 4 mm (n = 112) at the first GCF sampling site and second and third periodontal examinations at the same site (Steel–Dwass test)

Clinical and biochemical parameter	First periodontal examination	P1	Second periodontal examination	P2	Third periodontal examination	P3
PlI	0.22 ± 0.44	NS	0.24 ± 0.49	NS	0.34 ± 0.61	NS
PD (mm)	2.25 ± 0.79	NS	2.22 ± 0.75	NS	2.21 ± 0.78	NS
CAL (mm)	2.89 ± 1.35	NS	2.88 ± 1.35	NS	2.91 ± 1.48	NS
GI	0.49 ± 0.74	NS	0.56 ± 0.78	NS	0.57 ± 0.80	NS
BOP	0.08 ± 0.27	NS	0.18 ± 0.38	NS	0.23 ± 0.42	*
Hb (ng/pocket)	21.19 ± 49.80	**	4.03 ± 19.19	**	8.28 ± 6.65	**

*P1* First periodontal examination vs second periodontal examination

*P2* Second periodontal examination vs third periodontal examination

*P3* Third periodontal examination vs first periodontal examination

^*^p < 0.05, ^**^p < 0.01

When comparing the changes in clinical parameters and Hb values from the first periodontal disease examination to the third examination, the BOP value, in particular, showed a significant increase of 2.9 times from the first examination (p < 0.05), whereas the Hb value decreased significantly by 60.9% from the first examination to the third examination (p < 0.01) (Table 4).

Changes in the clinical parameter values or Hb values among the first, second, and third periodontal examinations in the PD ≥ 5 mm group.

The clinical parameter values of PlI, PD, CAL, and GI showed significant decreases (p < 0.01) from the first periodontal disease examination to the second examination, with reductions of 43.5%, 21.8%, 21.4%, and 29.7%, respectively. The BOP value decreased by 11.6% compared with the initial diagnosis of periodontal disease, but this decrease was not significant. In contrast, the Hb value decreased significantly by 76.4% compared with the initial one (p < 0.01) (Table 5).

**Table 5:** Comparison of periodontal examinations at a site of PD ≥ 5 mm (n = 79) at the first GCF sampling site and second and third periodontal examinations at the same site (Steel–Dwass test)

Clinical and biochemical parameter	First periodontal examination	P1	Second periodontal examination	P2	Third periodontal examination	P3
PlI	0.62 ± 0.54	**	0.35 ± 0.58	*	0.60 ± 0.66	NS
PD (mm)	5.46 ± 0.87	**	4.27 ± 1.49	NS	4.00 ± 1.37	NS
CAL (mm)	6.74 ± 1.76	**	5.30 ± 2.39	NS	5.47 ± 2.35	**
GI	1.28 ± 0.50	**	0.90 ± 0.65	NS	1.01 ± 0.79	**
BOP	0.43 ± 0.50	NS	0.38 ± 0.49	NS	0.50 ± 0.50	NS
Hb (ng/pocket)	86.41 ± 101.17	**	20.36 ± 43.64	**	11.74 ± 6.72	NS

*P1* First periodontal examination vs second periodontal examination

*P2* Second periodontal examination vs third periodontal examination

*P3* Third periodontal examination vs first periodontal examination

^*^p < 0.05, ^**^p < 0.01

Comparing the changes in the values of each parameter from the first periodontal examination to the third examination, the values of PlI, PD, CAL, and GI in the third periodontal examination decreased compared with the initial examination. The reductions of these values were 3.2%, 26.7%, 18.8%, and 21.1%. In contrast, the Hb level decreased significantly by 86.4% at the first examination. The decrease trends in PD, CAL, GI, and Hb levels from the first periodontal disease examination to the third examination over two years in the PD ≤ 5 mm group (79 periodontal sites) were similar to the changes in the same parameter values from the initial examination to the second over the one-year period. When comparing the changes in parameters from the second to the third examinations, the PD and Hb values decreased by 6.3% and 42.3% (p < 0.01), respectively, whereas the PlI, CAL, GI, and BOP values increased by 71.4% (p < 0.05), 3.2%, 12.2%, and 31.6%, respectively (Table 5).

The decreasing trends in PD, CAL, GI, and Hb values from the initial periodontal examination to the second or third examination were more pronounced in the PD ≥ 5 mm pocket group (Table 5) and all periodontal pockets (Table 2). However, these trends were not evident in the PD ≤ 4 mm pocket group (Table 4).

## Discussion

In this study, three periodontal examinations were performed in SPT patients: the first at baseline, the second examination one year after four SPTs, and the third two years after eight SPTs (Fig. 1). The periodontal examination included clinical parameters (PlI, PD, CAL, GI, and BOP) and a biochemical parameter (Hb levels in the GCF). In particular, Hb, indicating a history of bleeding, has been identified in GCF [12, 13], and we demonstrated that Hb in GCF is an important marker of the progression of periodontitis [14–16].

When the results of the first periodontal examination were compared with the results of the second examination one year after four SPTs, all other clinical and biochemical parameter values, except the BOP value, were similar to those of the first examination. Furthermore, when comparing the results of the first periodontal disease test with the results of the third periodontal disease test two years after the eight SPTs, the PD, CAL, and GI, but not PlI and BOP, and Hb values decreased (Table 2). Not only from the first examination to the second examination, but also from the first examination to the third examination, these decreases in the PD, CAL, GI, and Hb values suggest that SPT tends to improve periodontal disease to some extent. However, from the second to the third examinations, PD and Hb showed a decreasing trend, whereas other parameter values showed an increasing trend. This suggests that periodontal disease is markedly improved one year after the first periodontal examination, but there is not much change one year after the second periodontal examination.

These six parameters correlated with each other at each time point, not only at the first and second periodontal examinations, but also at the third examination (Table 3a-c). This suggests that the values of these parameters reflect the state of periodontal tissue to some extent.

Claffey et al. [22] have already discussed the condition of periodontal tissues in SPT patients by dividing them into groups with PD values ≤ 4 mm and ≥ 5 mm. In the present study as well, clinical parameters and Hb test values were categorized into two groups based on periodontal pocket depth (PD ≤ 4 mm and PD ≥ 5 mm), and each parameter was analyzed from the first periodontal examination to the second and third periodontal examinations (Fig. 1). These changes were then analyzed (Tables 4, 5).

In the PD ≤ 4 mm group (Table 4), of the five clinical parameters, only the value of PD decreased from the first examination to the second and third examinations. In addition, Hb levels in GCF, evidence of bleeding, were also significantly decreased. In contrast, from the second to the third examinations, all parameter values, except for PD, showed an increasing tendency. These results suggest that the inflamed periodontal tissues in the PD ≤ 4 mm group did not show any significant improvement even one year after the second examination.

However, in the PD ≥ 5 mm group (Table 5), all clinical parameters and Hb values, except for the BOP value, showed significant reductions from the first examination to the second examination. Furthermore, from the first examination to the third examination, the values of these parameters, except for BOP, also decreased. In addition, comparing these parameter values between the second and third examinations, the PD and Hb values decreased from the second examination to the third examination. This reduction in the Hb value was noticeable and significant. In the PD ≥ 5 mm group, unlike the PD ≤ 4 mm group, both PD and Hb values decreased sequentially and continuously from the first to the second and third examinations.

To summarize these findings, in unclassified pocket sites, the SPT conducted for one year between the first and second periodontal examinations decreased Hb values and most clinical parameter values, except for BOP. However, from the second to the third examinations, the PD ≥ 5 mm group, which had advanced periodontal disease, showed a significant decrease in PD and Hb values, unlike the unclassified group and the PD ≤ 4 mm group. These results suggest that periodontal tissues with deep pockets that are inflamed are more likely to improve through SPT than tissues with shallow pockets that are inflamed.

Badersten et al. reported that the rate of pocket reduction after scaling and root planing depends on the depth of the periodontal pocket [24, 25]. They suggested that the deeper the periodontal pockets, the greater the reduction rate of the pockets. The results of the present study were consistent with their results in that clinical parameters decreased, and pocket improvement was observed in deep pockets with a PD of 5 mm or greater after SPT intervention during the observation period.

In contrast, the changes in BOP test results from the first periodontal examination to the second and third examinations were different from the changes in other parameters. Lang et al. already reported that the negative predictive value of the BOP test is low [5]. We have reported a large number of negative results of the BOP test in severely inflamed periodontal tissue and classified the periodontal pockets by cut-off values for BOP and PD [7]. This phenomenon, peculiar to BOP, is thought to occur because the BOP test differs from other parameter tests in that it is a diagnostic method in which an external force is applied artificially to the periodontal tissue, and the presence or absence of bleeding is observed. In contrast, in the past, we have also reported that many periodontal tissues diagnosed BOP (-) are mildly damaged due to the presence of Hb in the GCF, despite being clinically stable [14, 15]. The results of the present study and the previous study [16] with long-term follow-up showed that changes in the Hb value in GCF correlated well with changes in clinical parameter values. However, the BOP test results were found to sometimes show no correlation with the results of other clinical parameter tests and the Hb test in GCF.

BOP and PD examinations using probes are tests that can evaluate the progression of periodontal disease by observing the vulnerability of periodontal tissues and attachment loss. The Hb test in GCF measures minute amounts of hemoglobin molecules in the blood within the periodontal pocket and can detect vascular damage in periodontal tissues caused by inflammation. Based on these findings, we believe that testing for Hb in GCF may be more sensitive in detecting the early stage of periodontal inflammation than testing for BOP. Therefore, we believe that the discrepancy in the results of the two tests is not only due to differences in testing methods, but also because the detection sensitivity of each test differs depending on the stage of progression of the periodontal disease being tested.

The usefulness of various markers in GCF component analysis has been reported, and clinical applications are expected [26]. Takeuchi et al. suggested that measuring the amounts of cytokines in GCF could more accurately reflect the condition of periodontal tissues, and that SPT could reduce cytokine levels [27]. Clinical application of a simple aspartate aminotransferase kit has also been reported [28]. Our developed hemoglobin test using the IC method is highly sensitive, simple, and rapid, with test results available in 15 min, making it suitable for chairside care.

Based on the above results, adding the Hb test in GCF to clinical parameter tests, such as the BOP test, will allow for a more accurate understanding of the state of inflamed periodontal tissues. We believe that the Hb test will contribute substantially to improving the accuracy of periodontal disease assessments after treatment.

## Data Availability

All data generated or analyzed during this study are included in this published article.
